# Artificial Intelligence and Precision Medicine: A New Frontier for the Treatment of Brain Tumors

**DOI:** 10.3390/life13010024

**Published:** 2022-12-22

**Authors:** Anil K. Philip, Betty Annie Samuel, Saurabh Bhatia, Shaden A. M. Khalifa, Hesham R. El-Seedi

**Affiliations:** 1 School of Pharmacy, University of Nizwa, Birkat Al Mouz, Nizwa 616, Oman; 2 Natural and Medical Science Research Center, University of Nizwa, Birkat Al Mouz, Nizwa 616, Oman; 3 Department of Molecular Biosciences, The Wenner-Gren Institute, Stockholm University, S-106 91 Stockholm, Sweden; 4 International Research Center for Food Nutrition and Safety, Jiangsu University, Zhenjiang 212013, China; 5 Pharmacognosy Group, Department of Pharmaceutical Biosciences, BMC, Uppsala University, SE-751 24 Uppsala, Sweden; 6 International Joint Research Laboratory of Intelligent Agriculture and Agri-Products Processing, Jiangsu Education Department, Jiangsu University, Nanjing 210024, China

**Keywords:** precision medicine, brain tumors, artificial intelligence, imaging technology, gene targeting, patient care

## Abstract

Brain tumors are a widespread and serious neurological phenomenon that can be life- threatening. The computing field has allowed for the development of artificial intelligence (AI), which can mimic the neural network of the human brain. One use of this technology has been to help researchers capture hidden, high-dimensional images of brain tumors. These images can provide new insights into the nature of brain tumors and help to improve treatment options. AI and precision medicine (PM) are converging to revolutionize healthcare. AI has the potential to improve cancer imaging interpretation in several ways, including more accurate tumor genotyping, more precise delineation of tumor volume, and better prediction of clinical outcomes. AI-assisted brain surgery can be an effective and safe option for treating brain tumors. This review discusses various AI and PM techniques that can be used in brain tumor treatment. These new techniques for the treatment of brain tumors, i.e., genomic profiling, microRNA panels, quantitative imaging, and radiomics, hold great promise for the future. However, there are challenges that must be overcome for these technologies to reach their full potential and improve healthcare.

## 1. Introduction

Brain tumors are a common form of cancer that can affect critical brain regions, often leading to cancer-related deaths (2.3%) [1]. According to the World Health Organization (WHO), glioblastoma, a tumor (grade IV) of the central nervous system (CNS), accounts for more than 60% of adult brain tumors [2]. Radiation therapy is a common treatment for glioblastoma, but it can have negative side effects, such as weakening the blood-brain barrier (BBB), making patients more susceptible to brain metastases [3]. For example, there have been reports of secondary brain tumors following radiation therapy for glioblastoma [4]. The process of metastasis is complex, involving multiple biological hurdles that tumor cells must overcome before they can establish themselves as metastatic lesions. Furthermore, the development of cancer treatments becomes more challenging when faced with intratumor heterogeneity, or the variation of genetic makeup within a tumor [5]. Use of brain-inspired computing could help simplify cancer management by providing a way to mimic the neural network of the human brain [6]. AI in healthcare can help to diagnose brain tumors [7] through the use of brain scans. In one study, AI was able to correctly identify 98% of brain tumors [8]. Machine learning algorithms applied to medical images can help to extract hidden features that human experts may not be able to discern. This can improve the accuracy of cancer diagnosis, prognosis, and treatment plans [9]. For example, in a study, deep learning technology was used on 1991 healthy samples and 12 cancer types showing an accuracy of 94.70% in identifying cancer [10].

The National Academy of Medicine states that AI technology in healthcare may offer benefits such as increased access to specialist healthcare and reduced human limitations [11]. A recent study found that AI-assisted surgery led to fewer complications and shorter hospital stays [12]. The use of AI in healthcare is growing rapidly. Global AI in the healthcare market is expected to grow to $150 billion by 2026 [13]. This growth is being driven by the increasing digitization of healthcare data [14], the improved ability of AI to analyze these data, and the potential benefits of AI in healthcare, such as early detection of disease, improved diagnosis, treatment recommendations, and personalized medicine [15]. AI is playing an increasingly important role in small-molecule drug discovery and development. AI is expected to play a major role in target selection, hit identification, and lead optimization in the near future [16]. For example, when eToxPred (a machine learning-based approach) was applied to estimate the toxicity and synthesis feasibility of small organic molecules, it showed accuracy as high as 72% [17]. Studies have shown that image-based diagnostic systems that use AI can often outperform clinicians. AI is able to more accurately recognize patterns and structures, which leads to more accurate diagnoses [18]. AI systems will improve with time based on real-life scenarios, feedback, and knowledge [19]. PM is an emerging medical model that uses a person’s genetic and molecular makeup to guide decisions about which medical treatments will work best for them [20]. The model has been used since the late 1990s to help select and customize treatments for individual patients with cancer [21]. The term “precision” in medicine and public health is becoming prevalent [22], and also involves medicines guided by molecules [23] or person-centred medicine [24], and provides evidence-based precise medical services [25]. PM is used in many fields, including clinical and preventive medicine [26,27]. PM identifies groups of patients benefiting from different treatment approaches using multiple data types [28], including data on a patient’s genes, environment, and lifestyle, enhancing patient health outcomes [29]. The information received from this approach can support PM in practice by helping to locate research related to a patient, and clinical trials [30].

Cancers are difficult to treat in patients due to a variety of reasons, including intra-tumoral heterogeneity and plasticity. Heterogeneity can make it difficult for drugs to target all of the cancer cells, and plasticity can allow cancer cells to become resistant to drugs over time [31,32]. Additionally, the presence of different subpopulations of cells within a tumor can cause the tumor to be dependent on these different cell groups for continued growth [33], and is therefore the main reason for treatment failures in cancer [32]. Intratumoral heterogeneity can be due to microenvironmental, genetic, and epigenetic factors [34]. Although we do not understand intratumoral heterogeneity very well [35], by understanding cellular subtypes and their development, cells can be targeted with PM [36]. Individualizing PM treatment must account for the patient’s cancer cells, genetic profile, and brain structure, especially when it comes to gliomas—a type of brain tumor that can be difficult to diagnose and treat. A new AI model created by researchers has the potential to be useful for diagnosing gliomas, as it can distinguish between urine samples from cancer patients and non-cancer patients. This model could be helpful for physicians in diagnosing and individualizing PM treatment for glioma patients [37,38]. Additionally, proteomics can provide a way to examine gliomas using fluid-based biomarkers [39]. This can help in understanding variations within this type of cancer, and potentially lead to better treatment options [40,41]. However, PM faces challenges due to the involvement of patient data, which includes data for disease, population diversity, and ethical reflections [42].

### 1.1. Molecular and Genomic Profiling of Brain Tumors and the Use of PM

Care of cancer patients can involve molecular profiling to choose the best treatment option [43] by finding gene mutations that may contribute to the disease, and targeting drugs that work best for that patient’s individual genetic makeup [44]. However, this method overlooks important molecular features that could have clinical significance [45], namely, prediction of drug-target group [46], molecular fingerprint representation [47], profile-to-cell line matchmaking [48], and drug-target interactions [49]. This method uses tissue biopsies to identify potential predictors of sensitivity and resistance [50]. If AI could correctly predict whether a tumor is benign or malignant, it could help doctors avoid performing unnecessary and potentially risky biopsies on patients. Studies have shown that AI can accurately predict whether a brain tumor is benign or malignant [51] with 95% accuracy [52], which would avoid the need for biopsy [53]. PM initiatives are a step forward in cancer treatment, but they come with challenges. Tumor tissue is difficult to work with, and other diagnostic and therapeutic methods are needed to overcome these challenges [54].

Molecular profiling of tumors can provide information on the specific genetic alterations present in the tumor, which can be used to guide treatment decisions. This has led to the development of targeted cancer therapies and the restructuring of clinical trials. Cancer is being treated at the molecular level by understanding the genetic profiles of tumors. The information can be used by clinicians to diagnose and treat cancer patients [55]. Cancer patients with brain tumors and metastases have not responded well to immunotherapy in the past [56]. A study found that immunotherapy and targeted therapy based on PM can treat brain metastases [57]. Genomic and molecular profiling of tumors reveals the function of tumor-derived genetic markers [58,59]. A study relating tumor biology with circulating tumor DNA (tDNA) levels was conducted and showed that patients with solid tumors had genomic alterations detected by plasma tDNA assay. The study supports the use of a genomic tumor profiling assay to detect genomic alterations in plasma tDNA from patients with active tumors [60]. Gene expression arrays (used in melanoma classifications) [61], and next-generation sequencing (NGS) are helping physicians determine how patients will respond to a particular therapy. These arrays and NGS will advance gene profiling technology to develop patient-specific treatments [62,63]. PM is very effective in treating some types of brain tumors, such as glioblastoma [64], using photodynamic therapy (PDT) [65]. Several approaches to overcome the challenges of implementing PM in glioblastoma have been reported, and integrated sequencing strategies have provided new insights into the molecular classifications and genomic landscape of several types of cancer [66]. The personalized PM service will use the microbiome, advanced clinical phenotyping (measurement of physical characteristics), diagnostics, advanced genomic imaging, and personalized genomics to enable PM [67,68,69]. Cancer immunotherapies targeting immune checkpoints are effective in enhancing immunity [70]. Immunotherapies for cancer patients can be improved by recognizing neoantigens and targeting them. Neoantigens are antigens that originate from somatic mutations. These mutated proteins located in tumor cells trigger a T-cell immune response [71]. PM can identify the type of brain tumor and the most effective treatment [72]. This may benefit patients with rare cancers that do, or cancers that do not, respond well to conventional treatments [73]. A study published in Nature reported that PM targets mutations in the IDH1 gene, which improves survival rates for patients with brain tumors [74]. Furthermore, PM targets genes for brain-specific [75] marker of metastasis [76] to provide an effective means to target cancer cells. Targeting reduces the risk of harming healthy brain tissue. With PM, patients will receive the best possible care and potential problems will be prevented [22].

Project management is a key part of developing new drugs and promoting the use of PMs in healthcare settings to improve patient care. PMs play a crucial role in many different settings, but their impact is especially significant when it comes to developing new treatments for specific types of cancer [77]. Deep learning is a new medical technology that is helping doctors save lives and improve patient outcomes. However, it is also creating new ethical dilemmas and raising questions about access to this information [78]. Deep learning is a branch of machine learning that employs algorithms to figure out high-level concepts from data. Machine learning, on the other hand, works on developing computer programs that have the ability to access data and interpret them. The biggest distinction between deep learning and machine learning is the level of abstraction it uses. Machine learning algorithms focus on low-level patterns present in data, while deep learning algorithms focus on high-level abstractions. There are several advantages of deep learning compared to traditional machine learning technologies. For example, deep learning typically results in fewer false positives per individual compared to traditional machine learning, indicating greater accuracy [79]. Researching human genetics has allowed for more precise cancer treatments through the usage of targeted drugs. For example, Zhao et al., describes an integrative analysis that indicates 13% of patients benefit from current targeted therapy based on gene mutation, and the proportion increases to 31% when drug repositioning is considered [80]. By identifying the gene responsible for cancer, researchers are able to target it with available drugs. However, a challenge that often arises is gene mutation, which can happen over the lifetime of a tumor. This makes it more difficult to predict which treatments would be the most effective. Another obstacle is that the gene mutation may be unique to an individual, making it difficult to create generalized detection tools [81].

**Figure 1:**
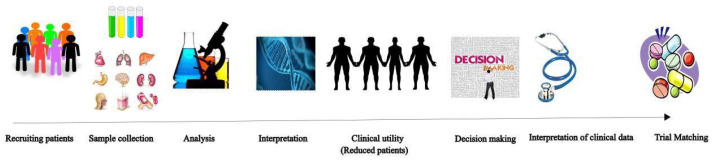
Attrition of patients during the process of recruiting the patient to trial matching. Adapted from reference [78], under creative common license.

It is challenging to recruit patients for PM studies due to the heterogeneity of the population. Informed consent is also an issue, as parents and patients may not fully understand the implications of participating in such a study [82]. Figure 1 [78] represents the attrition of patients during the process of genomic profiling to drug matching [83]. As can be seen, a large number of patients (30%) are lost between the initial stages of recruitment and the final stage of drug matching [17].

Informatics systems could simplify the recruitment process for clinical trials in PM by integrating genomic data and eligibility for clinical trials. This would allow for more accurate and efficient recruitment, as well as reducing the overall time and cost associated with clinical trials [84,85,86]. It is difficult to develop and study biomarkers due to the complexity of tumor heterogeneity. Another potential issue that can affect the precision of biomarkers is sampling bias [87]. Furthermore, due to the diversity of brain tumors, tissue banks are difficult to use for PM with respect to proper representation of the removed diseased area [88]. Thus, due to clonal development in brain tumors, it is important to look at many portions of the tumor [89]. The challenge of PM is predicting which tumors will respond to standard therapy and which will not. This may require the use of an angiogenesis inhibitor for tumors that do not respond to standard therapy [90,91]. Targeted drugs cannot change how a tumor forms, but they might be able to improve a person’s quality of life, according to some studies. However, there is disagreement among researchers as to whether targeted drugs are effective in cancer treatment, posing another obstacle to developing these drugs [92,93].

### 1.2. MicroRNA (miRNA) Panels as Markers in Brain Tumors

Brain tumors are often characterized by their expression of miRNAs [94]. The expression of miRNAs may provide information on tumor biology and the effect of therapeutic interventions [95]. MiRNAs can be tumor suppressors or promoters due to dysregulation in different types of cancer [96]. Additionally, miRNA can serve as a marker for non-invasive early detection of brain tumors [97]. A study carried out in 2170 patients with glioma and 1456 participants (healthy) in China supported earlier findings of the use of miRNAs as a marker for the detection of glioma [98]. A study, conducted in 31 people aged 61.1 to 62.9 years, analyzed miRNA expression levels in serum exosomes from cancer patients. The study found that hsa-miR-576-3p (small non-coding RNAs of 20–22 nucleotides) is a useful biomarker for predicting brain metastases in patients with breast cancer [99]. Some of these miRNAs may indicate a poor prognosis, while others may be associated with a better outcome [100]. MiRNAs may be useful markers for cancer diagnosis and monitoring. A prognostic [101] and diagnostic marker [102] can be found in up-regulation of miR-21. Various miRNA panels are potential markers for diagnosis, tumor grade, and prognosis [103]. Detection of diffuse glioma can be simplified by the use of miRNA. Furthermore, miRNA can differentiate primary CNS lymphoma from glioblastoma [104].

MiRNAs could be key targets for treating brain tumors, and may also help make tumors more sensitive to radiation therapy [105]. However, the role of miRNAs in biological processes (such as cell cycle, proliferation, apoptosis, and differentiation) needs to be carefully evaluated before using them as therapeutic targets [106]. Research is progressing on miRNA-based therapies for treating inflammatory diseases [107]. The safety of miRNA- based therapies can be ensured by developing nanocarrier-based platforms, which can also deliver miRNA-based therapies in a controlled and cell-specific manner [108]. Table 1 represents some relevant studies on the role of miRNAs in the oncogenesis of glioblastoma.

## 2. AI in Brain Tumor Imaging

The rapid growth of the AI industry, with substantial investment from technology firms, has outpaced expectations. Investing in AI projects [115,116], especially those related to medicine, is becoming increasingly popular [117]. The brain tumor diagnostics market is expected to grow rapidly in the coming years. The market was valued at $844.63 million in 2021 and is expected to reach $2476.14 million by 2028, growing at a compound annual growth rate (CAGR) of 16.6% from 2021 to 2028 [118]. AI helps doctors make better decisions by using complex computation and reasoning to help make decisions [11]. In medicine, AI is used for automated diagnostic procedures and treatments for patients [119]. An AI-based approach to cancer imaging can help improve tumor detection and characterization, as well as monitor the tumor’s response to treatment and check for early signs of cancer in other parts of the body [120].

Radiologists can find brain tumors quickly and effectively by using computer-aided diagnosis systems. These systems make use of supervised or unsupervised machine learning, transfer learning, or deep neural models (Xception model), all of which have been successful in the medical field thus far. AI and deep learning are expected to continue having breakthroughs in the future [121]. Molecular imaging provides the framework for new developments in the diagnosis of brain tumors. This technology allows for the visualization of molecular processes in the brain, which can provide critical information for diagnosis and treatment [122]. AI-based imaging algorithms (CXR-vision model, LIDC-IDRI model, LUNA16 model, and the CT-based volumetric analysis) have proven to be effective in diagnosing various cancers such as lung cancer [123], breast cancer (Mirai), and brain tumors [124]. Brain tumors have unique biological features which can be exploited by certain MRI sequences. T1-weighted images taken after gadolinium administration show enhanced areas where the blood-brain barrier has been breached, allowing gadolinium to enter the tumor from the intravascular space [125]. Orthogonal wavelet transforms and deep learning techniques are being used for the detection and classification of brain tumors [126]. A deep wavelet autoencoder (DWAE) model is being used to predict the location of a brain tumor based on the analysis of multimodal data such as MRI images, perfusion MRI images and PET scans. Furthermore, the combination of a DWAE model with a support vector machine helps the model learn the distribution of tumor volumes within the brain, and predict the classification of a tumor based on its volume [127].

Medical image segmentation is the process of partitioning a digital image into multiple segments (sets of pixels). The goal of segmentation is to simplify and/or change the representation of an image into something that is more meaningful and easier to analyze [128]. One important task in medical image processing is segmenting brain tumors from MRI scans. This helps doctors better understand the size and location of the tumor, as well as how it has changed over time. Deep learning-based segmentation of brain tumors is a popular method because it is automated and provides cutting-edge results [129]. For example, the deep capsule network (CapsNet) and latent-dynamic condition random field (LDCRF) can be used to segment brain tumors automatically. In contrast, a study of deep learning-based methods for detecting small tumors found that these methods were often inaccurate, resulting in misclassifications [130]. To better understand the human brain, doctors and researchers look for abnormalities. A study suggests that through finding these abnormalities and targeting them specifically, customized treatments for metastatic brain tumors (MBT) could be possible. This is in addition to the ability of MBT to provide a personalized diagnosis through its molecular expression profile. Furthermore, approximately 20–40% of MBT cases showed loss of DNA (MGMT) expression, highlighting the usefulness of this method. Another strength of MBT is that it expresses a variety of receptor and signal transduction molecules. This could potentially allow for individualized treatment using molecule-targeted drugs [131].

### 2.1. Quantitative Imaging of Brain Tumors

Magnetic resonance tomography (MRT), computed tomography (CT), and positron emission tomography (PET) are imaging techniques that are used to determine the location and size of a brain tumor. CT, MRT, and PET scans require contrast agents to produce clear images of the tumor [132]. The transcapillary transport of water-soluble compounds can be measured by PET methods in vivo. The PET method helps to understand brain tumors and their response to therapy. PET scans are also useful for determining the size and location of a tumor, which can aid in surgical planning [133]. PET scans are a valuable tool for studying the biology of brain tumors and could improve our understanding of brain tumors [134] by identifying areas of high metabolism that may be linked to tumor growth [135]. 18F-Fluorodeoxyglucose PET can be used to predict the prognosis of a patient and to distinguish nonmalignant from malignant lesions. 18F-Fluoroethyltyrosine, 11C-methionine, and 18F-L-3,4-dihydroxyphenylalanine are all high sensitivity markers that can be used to detect recurrent or residual cancer [136]. 3D-U-Net convolutional neural networks (CNNs) have been used to segment gliomas from 18-fluoroethyl-tyrosine PET scans. The CNNs showed high accuracy, with 78% positive prediction, 99% negative prediction, 88% sensitivity, and 99% specificity [137]. AMT-PET scanning identifies primary and metastatic brain tumors with 90% accuracy. This could advance the diagnosis and treatment of patients with metastatic brain tumors. Machine learning trained on MRIs predicts brain tumor outcomes better than established methods [138]. Brain MRI characterizes and visualizes the structure of interest in medical imaging. An AI-based automated method has been proposed that uses a classifier to identify and segment pathological tissue, such as tumors and atrophy, on brain MRI [139]. Aptamers are often used in PET research due to their high binding affinity and specificity. Aptamers can be easily labelled with radioisotopes, which allows researchers to study how molecules interact with each other [140]. Reduced fluorescence emission from brain tumors can be up to 50% lower than surrounding normal brain tissue, making tissue autofluorescence ideal for distinguishing between normal and tumor-affected brains. This technique is beneficial because it is noninvasive and can provide accurate results. Autofluorescence imaging has been used to successfully detect and map brain tumors in human patients [141].

Cancer is caused by uncontrolled cell division. Pathology is the key to understanding and diagnosing cancer [142,143]. Therefore, it is essential to obtain an accurate diagnosis to determine the best treatment plan [144]. That is why ultrasounds need image classification and object recognition algorithms that use deep learning to obtain precise results. These AI technologies include convolutional neural networks and recurrent neural networks. These algorithms have helped to examine medical images of various malignant neoplasms, such as brain tumors [145,146]. The rapid and noninvasive diagnosis of brain tumors is becoming increasingly popular [147]. This is in part due to the difficulty in diagnosing gliomas using MRI alone, and the possibility of irreversible errors. AI algorithms can help streamline this process and make it more accurate [148].

### 2.2. Radiomics in Brain Tumor Diagnosis

A precise diagnosis is essential for cancer treatment planning and predicting patient outcomes. Tumor classification and post-treatment response assessment are both improved when a precise diagnosis is made. In 2016, the WHO released an updated classification of brain tumors that integrated information on genetics [149]. Qualitative markers, such as tumor density and enhancement pattern, are used in conventional radiographic evaluation of tumors. In a study, it was shown that radiomics allowed for radiographic images to be digitally decoded to quantitative properties, which could then be used to distinguish between low-grade and high-grade gliomas [150]. Images fed into big data analytical tools provide information on tumor biology and therapeutic response [151]. Radiomics is the study of how medical images can be used to extract quantitative features that can be used to predict clinical outcomes [152]. Currently, there are no standard assessments of scientific integrity and clinical relevance [153]. Radiomics provides valuable information on tumor responses to therapy by incorporating AI into the glioblastoma multiforme (GBM) assessment of tumors using data from images. Radiomics uses sophisticated image analysis methods, such as diffusion and perfusion imaging, to provide accurate diagnosis and treatment of GBM [154]. Deep radiomic characteristics demonstrated markedly better precision (*p* < 0.05), with an AUC of 89.15%, compared to 78.07% for standard radiomic characteristics, for short and long-term survival prediction in patients with recurrent GBM [155]. Radiomics based on deep learning needs larger datasets to achieve better results due to the strong correlation between the extracted features and the input data. However, the limited availability of the dataset prevents radiomic implementation in many research areas. On the contrary, one technique that avoids this constraint is transfer learning. Transfer learning uses pre-trained neural networks for training interrelated purposes. For example, a neural network trained on imaging data to segment gliomas can be used for segmentation of brain metastases [149]. In one study, the researchers identified a signature of 11 radiographic characteristics to predict both survival and stratification in patients with newly diagnosed glioblastoma. The radiomic signature demonstrated improved performance over established radiological and clinical risk models [156]. Radiomics continues to integrate oncology, radiology, and machine learning, and is growing rapidly. In the future, radiomics will play an important role in precision diagnostics and oncology due to the ever-increasing clinical data and advances in machine learning methods. To improve the acceptability of radiomics, reproducibility and interpretability should be the focus. As per a study, radiomics can improve the accuracy of cancer diagnosis by up to 20% [157].

Radiogenomics (‘imaging genomics’) is a rapidly growing field that studies the relationship of genomic features of a disease to imaging biomarkers. In an earlier study, unsupervised learning algorithms and a knowledge based unsupervised fuzzy clustering approach, which is a type of algorithm, were discussed [125]. This new field is enabled by a three-way combination of textural, functional, and morphological signatures that are derived from high-throughput quantitative metrics extraction of MR images at the voxel level. The clinical application of radiogenomics is limited by the heterogeneity of brain tumors. Spatial and temporal heterogeneity can result in adverse clinical outcomes. Current cancer treatments work uniformly in tumors regardless of spatial or temporal variation in cancer cell behavior and survival. Performing whole tumor analysis by radiogenomics can address this limitation [158]. In response to modern chemotherapy/immunotherapy and radiation therapy, radiomics and radiogenomics show promise in providing accurate diagnosis, prediction of prognosis, and evaluation of tumor response.

### 2.3. Convolutional Neural Networks for Clinical Diagnostics

Different CNN architectures can be trained more quickly and accurately by using a combination of CNNs and stochastic gradient optimization algorithms. Emerging AI approaches, such as neural networks, deep learning, and CNN, help to retrieve important clinical data. These clinical data can be used for treatment planning and post-treatment monitoring [159]. By developing a fast and stable convergence method, it is possible to reduce the amount of time and resources needed to tune the momentum hyperparameters in popular CNN optimizers. This could improve the classification of images used in medical diagnostics [160]. CNNs can solve the problem of computer-aided diagnosis [161]. Ker et al., used a CNN to classify brain histological samples into high or low-grade glioma with 98% and 100% accuracy, respectively [162]. A demonstration by Havaei et al., showed that a CNN was 30 times faster and more precise than cutting-edge segmenting platforms [79]. Deep CNNs can extract significant features with high accuracy from GBM histopathology images [163]. With continuous improvements in the prediction of the accuracy of the system, deep CNNs can be a powerful clinical tool for the early detection and management of GBM. Raman spectroscopy probes have recently been used to find brain tumors in real time during surgery. This technology can detect diseased tissues up to a millimeter deep because it collects high-quality signals rapidly [164]. This ability is beneficial in neurosurgical procedures [165]. It can detect tumor margins and give surgeons immediate feedback on whether tumor cells are still present [166]. Clinical data shows that tumor classification based on a combination of 3D CNN characteristics is highly accurate and can improve clinical outcomes by facilitating the selection of the most appropriate treatment regimen for patients [167].

## 3. Future of AI in Brain Tumor

Although AI technologies have changed diagnostic radiology a lot, there are many areas that still need improvement. These areas include AI applied to detect, segment, and classify brain tumors, which would make patient care better [168]. The recent integration of an AI system into the clinical workflow indicates that AI can be used to improve clinical care [169]. AI can be used for early diagnosis of gliomas in the absence of visual contrast; however, there is currently a lack of high-quality image data which limits its potential. A future step in AI development and imaging technologies is to recognize pre-metastatic niches. The early detection of these niches provides an accurate assessment of a patient’s probability to develop metastatic or micrometastatic disease. AI applications can be divided into two categories: (1) upstream AI applications that concern operational analytics, and (2) downstream AI applications that are focused on the imaging data themselves [170]. Improvement in the accuracy and efficiency of AI applications is possible by combining different types of annotations. A distinction like this can be limiting because it prevents different annotation types from being combined. This will increase the interest in and supervision of medical image analysis [171]. AI in neurology has a promising future because it has the potential to predict seizures [172] and grade brain tumors [173]. The model was significantly improved when it was used to segment brain tumors on MR images. This was done by multitasking with global labels and local annotations [174]. The researchers showed that AI algorithms can accurately segment intracranial hemorrhages on brain CT images and measure hemorrhage volumes. The device could be used to detect and measure head and neck vascular tumors or malformations [175]. AI has the potential to provide significant advances in the accurate interpretation of cancer imaging, including extrapolation of the tumor genotype, volumetric delineation over time, and prediction of clinical outcome based on the phenotype of its radiographic appearance [151].

## 4. Challenges for Using AI with Brain Tumors

Brain gliomas are one of the most difficult cancers to detect and classify [176]. Gliomas are often small and difficult to see on imaging tests, and their symptoms can be vague and mimic other conditions. Deep learning and machine learning have the potential to change the diagnosis of glioma in the future [147]. AI is becoming increasingly popular in smart healthcare [177,178]. A significant barrier healthcare providers face is the lack of resources and investment in information technology. Another is the lack of training on how to use big data. Big data can be analyzed using sophisticated methods that are designed to handle the volume, variety, and velocity of big data. An intelligent tutoring system or process-oriented e-learning system can help train personnel in big data. Additionally, there are concerns about data security and privacy [179], for example access control models and privacy-preserving protocols. Centralized AI is being used by healthcare providers to overcome the issue of not having enough data to train machine learning models. However, it may be difficult to transfer sensitive patient information from hospitals to these processing centers, as this requires a lot of time and resources. This could then limit inter-center research cooperation [179]. Instead, healthcare institutions could use federated learning to collaborate with each other. Federated learning is a machine learning technique that trains an algorithm across multiple decentralized edge devices or servers holding local data. This offers a way to unlock information without anyone seeing or touching the data. Federated learning would allow different healthcare providers to keep their sensitive data private while still being able to train machine learning models on shared data. Despite some progress, brain tumor research still has many limitations. A challenge has been grading tumors by human interpretation of images. This process involves some subjectivity in the classification of tumor grade based on morphology and the person interpreting the images. Therefore, a more accurate diagnosis can be sought through an automated image analytic process, which will assist in a quantitatively objective classification process for brain tumors [180]. Emerging AI methods have shown great promise for medical imaging in radiology [149].

## 5. Conclusions

The use of AI as a support tool in cancer intervention and prevention strategies has shown promising results. AI-assisted brain tumor surgery can result in safer and more effective treatment. By integrating clinical, radiological, and molecular markers, AI has the potential to significantly improve patient care. Recent developments in PM have emphasized targeted therapies and customized treatment techniques. Although the large-scale implementation of AI and PM in brain tumor treatment is facing challenges, the tremendous pace at which they are getting developed holds a great promise to remarkable progress in the outcomes.

## Figures and Tables

**Table 1:** Relevant studies in relation to the role of miRNAs in the oncogenesis of malignant primary brain tumors.

Tumor Type	miRNA	Gene-Target	Biological Function		Signalling Pathway	References
Glioblastoma	miR-128-3p	platelet-derived growth factor alpha receptor	promotes glioblastoma	Down	receptor tyrosine kinase	[109]
Glioblastoma	miR-218	hypoxia-inducible factor 2 alpha	promotes glioblastoma	Down	receptor tyrosine kinase	[110]
Glioblastoma	miR-95	Hepatocyte Growth Factor and Mitogen-Activated Protein Kinase Kinase 3	improved clinical outcome in the neural subtype	Down	Signal transducer and activator of transcription 3	[111]
Glioblastoma	miR-21	Integrin b8 [112]	improved clinical outcome in the neural subtype	Up	Signal Transducer and Activator of Transcription [113]	[111]
Glioblastoma	miR-381	lymphoid enhancer-	Inhibits metastases	Down	Wnt	[114]
